# Tumor Necrosis Factor-Alpha Modulates Expression of Genes Involved in Cytokines and Chemokine Pathways in Proliferative Myoblast Cells

**DOI:** 10.3390/cells13131161

**Published:** 2024-07-08

**Authors:** Angela María Alvarez, Carlos Eduardo Madureira Trufen, Marcus Vinicius Buri, Marcela Bego Nering de Sousa, Francisco Ivanio Arruda-Alves, Flavio Lichtenstein, Ursula Castro de Oliveira, Inácio de Loiola Meirelles Junqueira-de-Azevedo, Catarina Teixeira, Vanessa Moreira

**Affiliations:** 1 Centre of Excellence in New Target Discovery (CENTD), Butantan Institute, Sao Paulo 05503-900, SP, Brazil; angela.alvarezg@udea.edu.co (A.M.A.); carlos.trufen@gmail.com (C.E.M.T.); marcus.buri@butantan.gov.br (M.V.B.); francisco@pulchercode.com (F.I.A.-A.); flavio.lichtenstein@butantan.gov.br (F.L.); 2 Reproduction Group, Pharmacy Department, School of Pharmaceutical and Food Sciences, University of Antioquia—UdeA, Medellín 050010, Colombia; 3 Departamento de Farmacologia, Escola Paulista de Medicina, Universidade Federal de Sao Paulo, Sao Paulo 04044-020, SP, Brazil; marcela.nering@unifesp.br; 4 Czech Centre for Phenogenomics, Institute of Molecular Genetics of the Czech Academy of Sciences, v.i, 252 50 Vestec, Czech Republic; 5 Laboratório de Toxinologia Aplicada, Center of Toxins, Immune-Response and Cell Signaling (CeTICS), Butantan Institute, Sao Paulo 05503-900, SP, Brazil; ursula.castrolive@gmail.com (U.C.d.O.); ijuncaze@butantan.gov.br (I.d.L.M.J.-d.-A.); 6 Laboratório de Farmacologia, Butantan Institute, Sao Paulo 05503-900, SP, Brazil

**Keywords:** muscle regeneration, inflammation, cytokine

## Abstract

Skeletal muscle regeneration after injury is a complex process involving inflammatory signaling and myoblast activation. Pro-inflammatory cytokines like tumor necrosis factor-alpha (TNF-α) are key mediators, but their effects on gene expression in proliferating myoblasts are unclear. We performed the RNA sequencing of TNF-α treated C2C12 myoblasts to elucidate the signaling pathways and gene networks regulated by TNF-α during myoblast proliferation. The TNF-α (10 ng/mL) treatment of C2C12 cells led to 958 differentially expressed genes compared to the controls. Pathway analysis revealed significant regulation of TNF-α signaling, along with the chemokine and IL-17 pathways. Key upregulated genes included cytokines (e.g., IL-6), chemokines (e.g., CCL7), and matrix metalloproteinases (MMPs). TNF-α increased myogenic factor 5 (Myf5) but decreased MyoD protein levels and stimulated the release of MMP-9, MMP-10, and MMP-13. TNF-α also upregulates versican and myostatin mRNA. Overall, our study demonstrates the TNF-α modulation of distinct gene expression patterns and signaling pathways that likely contribute to enhanced myoblast proliferation while suppressing premature differentiation after muscle injury. Elucidating the mechanisms involved in skeletal muscle regeneration can aid in the development of regeneration-enhancing therapeutics.

## 1. Introduction

The skeletal muscle has a remarkable regenerative capacity, which gradually declines with aging or muscle diseases, resulting in compromised muscle function [1,2]. Muscle regeneration involves a complex series of highly coordinated events, including satellite cell activation which turns into myoblasts [3]. After skeletal muscle injury, the differentiation of myoblasts into myotubes and maturation into myofibers is essential for muscle repair [1,2]. Dysregulation of these processes can result in pathologies, including a suite of muscular dystrophies, cachexia, and sarcopenia [4]. Despite significant research efforts, the complex signaling mechanisms underlying skeletal myogenesis are still not fully understood. The initiation of the myogenic program is a critical step in skeletal muscle regeneration, which requires chromatin remodeling in myogenic cells to enable the transcriptional activation of myogenic target genes [5]. Intramuscular inflammatory signaling plays a critical role in mediating the regenerative response to muscle fiber damage [6]. A transient increase in local inflammatory signaling triggers a pro-myogenic signaling cascade that leads to the repair, remodeling, and maintenance of healthy muscle tissue [7]. However, an excessive or persistent inflammatory response can prevent myogenesis and limit recovery. Cytokines released during the early inflammatory response strongly influence the normal progression of the proliferative stage by promoting both muscle growth and muscle loss [8,9,10].

The cytokine TNF-α constitutes a key mediator of the initial inflammatory response in the process of skeletal muscle regeneration [11], triggering biological processes, including the activation of white blood cells, cell survival and proliferation, and apoptosis [12]. TNF-α is primarily synthesized by macrophages, although it is expressed by different cell types, including skeletal muscle cells [13,14,15]. It transduces its activity via a family of glycoprotein receptors, of which tumor necrosis factor receptors type 1 and 2 (TNFR1 and TNFR 2) are the most studied [16,17,18]. TNF-receptor engagement by the cytokine leads to the recruitment of receptor-specific adaptor proteins, which in turn activate a cascade of protein kinases and cell-specific downstream transcription factors, with Nuclear Factor-κB being the most widely studied in different cell types [19]. TNF-α levels rise substantially in injured muscle due to its increased synthesis by injured myofibrils and infiltrating inflammatory cells [20]. High TNF-α levels found in cultures during the early phase of C2C12 myoblast differentiation have been positively associated with regeneration, as inhibition of this cytokine prevented muscle repair [21]. The early increase in TNF-α levels is implicated in myogenic cell migration to the site of injury [22], myoblast proliferation, and the inhibition of myogenic differentiation [23,24,25]. Injured muscle fibers also show an increased expression of TNF-α receptors, which regulate the exiting of the cell cycle and the initiation of myogenic differentiation [26].

The effects of TNF-α in myogenesis appear to be highly associated with its concentrations at the site of muscle injury. Higher concentrations of recombinant TNF-α (≥0.5 ng/mL) inhibit the progression of myogenesis, while low concentrations of TNF-α (0.05 ng/mL) achieve enhanced proliferation and differentiation of satellite cells in culture by activating p38 MAPK signaling, thus leading to muscle repair [7,26,27]. In this context, we recently demonstrated that low concentrations of TNF-α increased the proliferation of C2C12 satellite-derived cells via the release of IL-6 and IL-15, a myokine involved in the growth and repair [12] of damaged myofibers but regulated the differentiation process by decreasing the nuclear expression of the transcription factors Pax7, MyoD, and myogenin in C2C12 myoblast cells. 

The relevance and molecular mechanisms of TNF-α involvement in muscle regeneration remain unclear. Thus, identifying the key genes that are differentially regulated by TNF-α and their target pathways in myoblasts has become crucial to better understanding the intrinsic mechanisms of TNF-α involved in myogenesis. Studies involving gene expression profiling using skeletal muscle myotubes in culture have identified novel genes that mediate the catabolic effects of TNF-α and the loss of skeletal muscle mass [28]. However, the effects of low concentration of TNF-α on global gene expression and intracellular pathways associated with regeneration in myoblasts are still unknown. 

In this study, we use a global gene expression approach to identify key genes and pathways that are significantly regulated by low-concentration TNF-α in C2C12 myoblasts. Our results demonstrate that TNF-α regulates the expression of several genes and pathways related to its stimulatory action on the regeneration process of skeletal muscle. Furthermore, our study provides the first evidence that TNF-α activates specific pathways in myoblasts. These results may contribute to the development of new therapeutic interventions to increase the muscle regeneration and attenuate muscle atrophy after muscle injury.

## 2. Materials and Methods

### 2.1. Cell Culture and TNF-α Treatment

The mouse myoblast cell line C2C12 (American Type Culture Collection, Manassas, VA, USA) was grown up to 80% confluence in Dulbecco’s modified Eagle’s medium (DMEM) (Gibco, Grand Island, NY, USA) supplemented with 10% heat-inactivated fetal bovine serum (FBS), 1% L-glutamine (Gibco) and 1% penicillin–streptomycin (Gibco) at 37 °C in a 5% CO_2_ incubator. The cells were plated at 2 × 10^4^ cells per well in six-well plates (Corning, Corning, NY, USA) previously coated with 2% gelatin (Sigma-Aldrich, Saint Louis, MO, USA). After 48 h in DMEM-10%, the medium was replaced with fresh DMEM-10%. The cells were then incubated with 10 ng/mL TNF-α (Bio Legend, San Diego, CA, USA) or DMEM-10% only (as an untreated control) for 24 h.

### 2.2. RNA-Seq Analysis

#### 2.2.1. RNA Extraction and Quality Control

The myoblast cells were treated with 10 ng/mL TNF-α and maintained in culture for 24 h. We extracted total mRNA from cultured cells using the RNeasy mini kit (Qiagen, Hilden, Germany) according to manufacturer instructions and assessed the mRNA concentration and quality using UV spectrophotometry (NanoDrop, Thermo Fisher Scientific, Waltham, MA, USA).

#### 2.2.2. Transcriptome Sequencing and Assembly

The TruSeq RNA library kit v2.0 (Illumina, San Diego, CA, USA) was used to convert mRNA into cDNA libraries. Validation for size was performed on an Agilent Bioanalyzer 2100 (Agilent Technologies, Waldbronn, Germany) with final sequencing products, which were sequenced with a 2 × 201 bp paired-end sequencing module on an Illumina HiSeq 1500 (Illumina). The preprocessing of raw sequence reads included the removal of viral PhiX control libraries with Bowtie2 version 2.2.5 [29], adapters, and low-quality bases with the Trimmomatic software version 0.36 [30]. The quality of raw reads was assessed through the FastQC software v0.11.7 [31].

#### 2.2.3. Transcriptome Annotation

The mouse annotation file was obtained from the Ensembl genome browser 90, corresponding to the Genome Reference Consortium Mouse Build 38. The gene symbols were gathered from the Mouse Genome Informatics (MGI) [32], with their corresponding descriptions obtained from the biomarRt package [33,34]. Paired-end reads with sufficient quality and trimmed barcodes were aligned to the GRCm38 using FeatureCounts [35]. This method produced a matrix with samples as columns and transcripts as rows, along with their corresponding read counts. Transcripts with less than 1 TPM per library were considered low expressed and discarded.

### 2.3. Protein Expression by Western Blotting

We obtained protein lysates from myoblast cells that were treated with 10 ng/mL TNF-α and maintained in culture for 24 h. The supernatant was drained, and cell lysates were prepared with Laemmli buffer. An equal amount of protein extracts was resolved on 12% sodium dodecyl sulfate–polyacrylamide gel electrophoresis under reduced conditions. Separated proteins were transferred to nitrocellulose membranes (GE Healthcare, Chicago, IL, USA), and the membranes were then blocked with 5% non-fat powdered milk. The membranes were incubated overnight at 4 °C with primary antibodies Myf5, MyoD, and TIMP-1 rabbit anti-mouse or mouse anti-mouse (Santa Cruz Technology, Dallas, TX, USA; Thermo Fisher Scientific). Then, the membranes were washed and incubated with donkey anti-rabbit or sheep anti-mouse secondary antibodies conjugated with horseradish peroxidase (HRP) (GE Healthcare). Peroxidase-conjugated antibodies were detected by chemiluminescence using Immobilon Western (Millipore, Billerica, MA, USA). The constitutive protein β-actin was used as an internal loading control, detected by an anti-mouse antibody (Sigma-Aldrich) and HRP-conjugated sheep anti-mouse secondary antibody (GE Healthcare). The intensity of the bands was quantified by densitometry using Image J (Version 1.54j) (NIH, Bethesda, MD, USA), and protein expression was calculated as a ratio to β-actin control.

### 2.4. Metalloproteinases and Interleukin-6 (IL-6) Release by Enzyme-Linked Immunosorbent Assay (ELISA)

Myoblast cells were treated with 10 ng/mL TNF-α and cultured for 24 h. Cell-free supernatants were collected and stored at −80 °C until analysis. Enzyme-linked immunosorbent assays (ELISA) were conducted to measure the concentration of matrix metalloproteinases (MMP) 9, 10, and 13 (Bioassay Technology Laboratory, Jiaxing, China), as well as IL-6 (Thermo Fisher Scientific, Waltham, MA, USA), following the manufacturer’s instructions. Samples were incubated for 1 h with specific antibodies and streptavidin-HRP conjugates. After the addition of the substrate solution, the plates were read at 450 nm using a spectrophotometer (SpectraMax, Molecular Devices, San Jose, CA, USA). The levels of each MMP and IL-6 in the culture supernatant were interpolated from the standard curves.

### 2.5. High-Content Screening (HCS) Analysis

Following a 24-h incubation of C2C12 proliferative cells with TNF-α (10 ng/mL), the cells were fixed with 4% paraformaldehyde, permeabilized, and blocked using 1% bovine serum albumin. Subsequently, anti-NF-κB (p65) primary antibodies (Cell Signaling Technology, Danvers, MA, USA) were applied and allowed to incubate overnight at 4 °C. After thorough washing, the cells were stained with Hoechst DNA-specific dye (Thermo Fisher Scientific) and incubated with goat anti-rabbit IgG secondary antibody Alexa Fluor 467 (Thermo Fisher Scientific). Additionally, the cytoskeleton of C2C12 cells was labeled with cytoskeleton F-actin Alexa Fluor 488 Phalloidin (Thermo Fisher Scientific). High-content image analysis was then conducted using MetaXpress^®^ High-Content Image Acquisition and Analysis Software version 6 (Molecular Devices), capturing 16 images/well at 20× magnification. The quantitative data represent the average fluorescence intensity of the p65 protein, which was corrected using the respective secondary antibody control.

### 2.6. Gene Expression by Real-Time Reverse Transcription PCR (RT-qPCR)

Myoblast cells were treated with 10 ng/mL TNF-α for 24 h. Total mRNA was extracted as previously described and assessed for mRNA concentration and quality via spectrophotometry (NanoDrop). The mRNA was reverse transcribed into cDNA using the QuantiNova Reverse Transcription Kit (Qiagen) according to manufacturer instructions. The QuantiNova SYBR Green PCR (Qiagen) was employed for quantitative real-time PCR (RT-qPCR) with gene-specific primer pairs for myostatin (Thermo Fisher Scientific), versican, and GAPDH (Exxtend, São Paulo, Brazil). The expression levels of the genes of interest were determined using the QuantStudio real-time PCR system (Thermo Fisher Scientific). We used the 2-ΔΔCT method [36] to calculate the fold change cDNAs relative to the untreated control, with GAPDH serving as the housekeeping gene of choice. Primer sequences were designed using the primer design tool from NCBI [37] and are listed in Table 1.

### 2.7. Statistical Analysis

#### 2.7.1. Differential Gene Expression

Differential expression analysis was performed using the R programming language [38] with the edgeR package [39]. Transcripts with a low expression were filtered out, and library sizes were normalized with TMM using the calcNormFactors function [40]. We calculated the dispersion estimates using the estimateDisp [41] function, followed by fitting a linear model using the glmFit function [42]. Finally, we performed a Likelihood-Ratio test using the glmLRT function. A table with the results was created using the topTags function. Multiple hypothesis testing was corrected using the False Discovery Rate (FDR) [43]. From this table, the differentially expressed genes (hereafter referred to as DEGs) were filtered based on absolute log_2_ fold change (LFC) ≥ 1 and FDR < 0.05. A volcano plot was generated using the ggplot2 [44] package to compare the TNF-treated samples with the untreated control. 

#### 2.7.2. Gene Set Enrichment Analysis 

Using DEGs ranked by LFC as input, we performed a gene set enrichment analysis (GSEA) using the GSEA function from the clusterProfiler package [45]. We accessed the publicly available databases KEGG [46], Gene Ontology [47], and Reactome from the Enrichr [48] website and plotted the corresponding figures using the gseaplot2 function from the enrichplot [49] package.

#### 2.7.3. Validation Experiments

The results obtained from Western blotting, ELISA and RT-qPCR analysis were performed at least three times. Data are expressed as the mean ± standard error of the mean (SEM). According to the data distribution, assessed by the Shapiro–Wilk test [50], group differences were evaluated using the parametric Student *t*-test or the non-parametric Mann–Whitney test. Statistical significance was determined as *p* < 0.05. Statistical analyses were conducted using GraphPad Prism 8.0.2 (GraphPad Software, La Joya, CA, USA).

## 3. Results

### 3.1. Transcriptomic Results and Differential Expression

**Figure 1:**
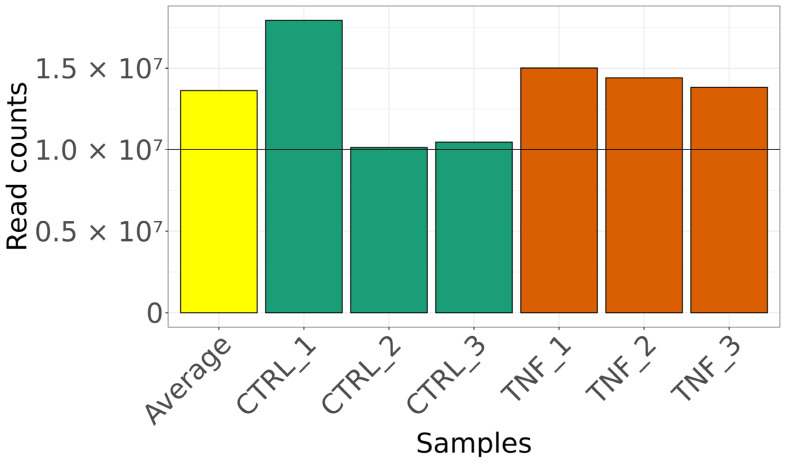
Read counts per each library in the RNA-Seq. On the left, colored yellow, is the average of read counts per library. We found 958 differentially expressed genes (DEGs) in samples treated with TNF-α compared to untreated samples, used as control (Supplementary Table S1, Figure 2). Our results agree with other studies reporting that TNF-α modulates the expression of several genes in myoblast cells, using other technologies such as microarray-based gene expression [28,51].

From the RNA-Seq experiments, the sequenced libraries yielded enough read mapping in each sample (Figure 1). With the chosen criterion for feature selection, 14,221 out of 52,636 transcripts were filtered for the further calculation of differential expression analysis.

**Figure 2:**
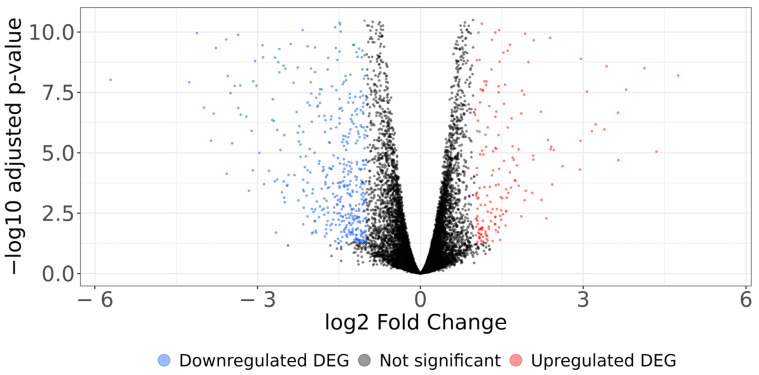
Volcano plot illustrating the differential gene expression between TNF-α treated and untreated samples. The *x* axis displays the log_2_ fold change (LFC) or relative abundance, and the *y* axis presents the adjusted *p*-value (−log_10_) obtained through the FDR algorithm. Upregulated genes are red dots on the right side, whereas downregulated genes are green dots on the left, and genes with no significant change are black dots.

### 3.2. Pathway Enrichment Analysis 

**Figure 3:**
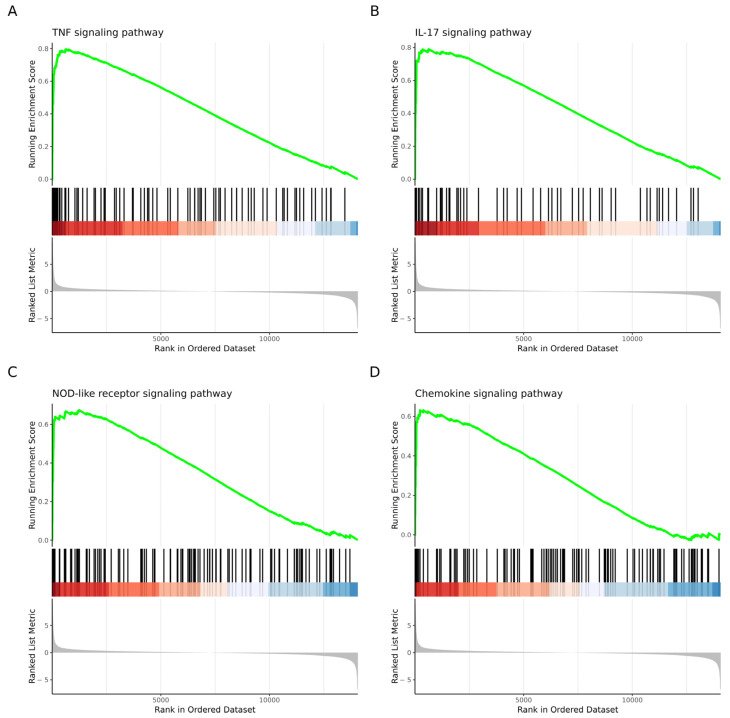
GSEA plot showing the most significantly enriched pathways using the KEGG database. The most significantly enriched pathways were (**A**) the first most significantly enriched pathway was TNF signaling pathway; (**B**) the second was IL-17 signaling pathway; (**C**) the third was NOD-like receptor pathway; and (**D**) the fourth was chemokine signaling pathway.

Using the list of DEGs, we found 85 enriched pathways in the KEGG database. The most statistically significant pathways were “TNF signaling”, “IL-17 signaling”, “NOD-like receptor”, and “chemokine signaling” (Figure 3 and Table 2). The Supplementary Table S2 contains information on all the enriched pathways. 

### 3.3. TNF-α Modulates Distinct Gene Networks in Myoblast Cells

As described above, TNF-α significantly modulated the expression of several genes in myoblast cells (Supplementary Table S1). Among the most up-regulated genes with a higher LFC, we found several chemokines, cytokines, myokines, and adhesion molecules. Chemokines such as CCL5 (logFC 8.5), CCL2 (logFC 6.61), CXCL3 (logFC 6.13), CCL7 (logFC 4.54), and CXCL16 (logFC 4.44) were among those identified. With respect to their involvement in myoblast function, CCL5, CCL2, and CCL7 promote myoblast proliferation, while CXCL3 contributes to muscle cell differentiation [52,53,54,55]. CXCL16 also promotes muscle regeneration through the regulation of neutrophil and macrophage infiltration into injured muscle [56]. Cytokines such as IL-1α (logFC 6.08), LIF (logFC 3.23), and IL-6 (logFC 2.96) have been described as key factors for stimulating myoblast proliferation [57,58], while cytokine receptors such as IL-2 receptor gamma (IL2-Rγ) (logFC 4.74) and IL-1 receptor-like 1 (IL1rl1) (logFC 4.41) are less studied in the skeletal muscle cell differentiation process. IL2-Rγ is a shared receptor (CD132) for IL-2, IL-15, and IL-17 [59]. IL-15 is a specific cytokine produced by skeletal muscle (myokine), whose production in myoblast cells is, in turn, stimulated by TNF-α [12].

Among the down-regulated genes identified were the chemokine receptor CXCR4 (logFC −2.72), the cytokine IL-33 (logFC −3.32), the cytokine receptor IL-23R (logFC −3.31), and the growth factor IGF-1 (logFC −5.43). CXCR4, which plays a role in skeletal muscle development, has recently been described as a regulator of satellite cell activation [60]. IL-33, which is released following cellular damage, is classified as an alarmin and has been associated with musculoskeletal pathologies [61]. The proinflammatory cytokine IL-23 belongs to the IL-12 family, which signals through the paired chains of five receptors, including IL-23R [62]. This pathway is critical in the creation of autoimmune conditions, as it stimulates the production of other pro-inflammatory cytokines in Th17 cells [63]. Finally, insulin-like growth factor 1 (IGF1) regulates both anabolic and catabolic pathways in skeletal muscle [64].

**Figure 4:**
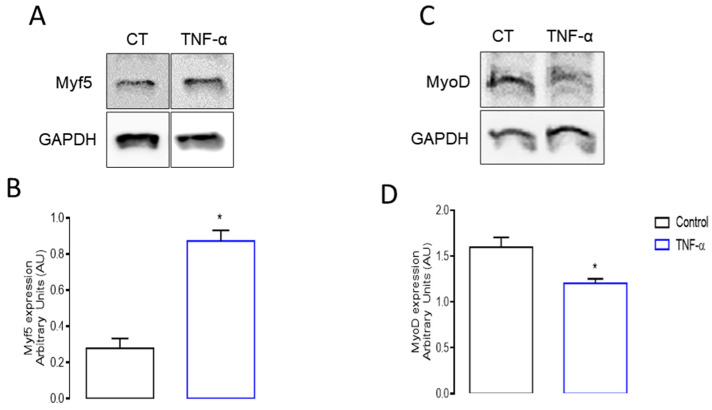
Protein expression of myogenic regulatory factors Myf5 and MyoD. The expressions of Myf5 and MyoD were assessed by Western blotting in proliferative myoblast cells stimulated with TNF-α for 24 h. Representative blots are presented for both (**A**) Myf5 and (**C**) MyoD protein expressions. TNF-α induced an (**B**) increase in Myf5 expression, while in contrast, (**D**) MyoD expression decreased after 24 h of TNF-α incubation (* *p* < 0.05 from unpaired *t*-test, *n* = 6).

To validate our findings and enhance our understanding of the effects of TNF-α on proliferating C2C12 cells, we performed Western blot, qPCR, and ELISA assays to assess the expression and release of the selected proteins. Myogenic differentiation begins as progenitor cells upregulate myogenic regulatory factors (MRFs), including Myf5 and MyoD. These two transcription factors are essential to regulate regenerative muscle-specific gene expression [65]. According to the transcriptome, the transcript of Myf5 (see Table S1) was barely reduced (logFC −1.65) in TNF-α-treated proliferative myoblasts. On the other hand, the incubation of C2C12 cells with TNF-α promoted a significant increase in the protein level of 68% of Myf-5 (*p* < 0.05) when compared to the control cell group (Figure 4A,B). We also assessed TNF-α-induced MyoD protein expression and our results showed a significant decrease (*p* < 0.05) in the protein levels by 25% compared to the respective cells cultivated with the culture medium only (Figure 4C,D). 

**Figure 5:**
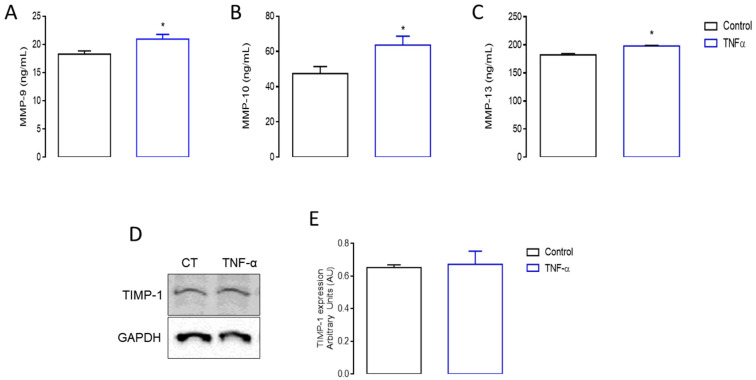
Matrix metalloproteinases (MMPs) production and tissue inhibitor of metalloproteinase 1 (TIMP-1) expression. MMP-9, 10, and 13 production was measured by ELISA in proliferative myoblast cells stimulated with TNF-α for 24 h. TNF-α treatment resulted in the increased production of (**A**) MMP-9, (**B**) MMP-10, and (**C**) MMP-13. Additionally, the TIMP-1 expression was examined by Western blotting in myoblast cells stimulated with TNF-α for 24 h. Representative blots are shown for TIMP-1 (**D**), and its densitometric analysis (**E**) (* *p* < 0.05 from unpaired *t*-test, *n* = 6).

According to Table S1, we identified increased gene induction in C2C12 cells of matrix metalloproteinases (MMP) by TNF-α, which is a family of proteolytic enzymes that increase their activity during remodeling processes or tissue inflammation [66]. Among them, we highlight MMP-13 (logFC 8.84), MMP-9 (logFC 7.72) and MMP-10 (logFC 7.14). To validate these results, the production of these MMPs was quantified in the supernatant of proliferative C2C12 myoblasts under the action of TNF-α. Our results in Figure 5A–C confirmed that TNF-α significantly (*p* < 0.05) stimulated the secretion of identified MMPs (MMP-9: 21.0 ± 0.8; MMP-10: 63.3 ± 5.1; MMP-13: 198.0 ± 1.1 ng/mL), when compared to the respective control cell groups (MMP-9: 18.3 ± 0.6; MMP-10: 47.4 ± 4.0; MMP-13: 182.1 ± 2.3 ng/mL). MMP activity is regulated by the tissue inhibitors of the metalloproteinases (TIMPs), which reversibly inhibit MMPs to prevent excessive and deleterious tissue degradation [67]. According to Figure 5D,E we looked for the TIMP-1 protein expression and found that it did not change in our experimental condition after TNF-α treatment (*p* < 0.05).

**Figure 6:**
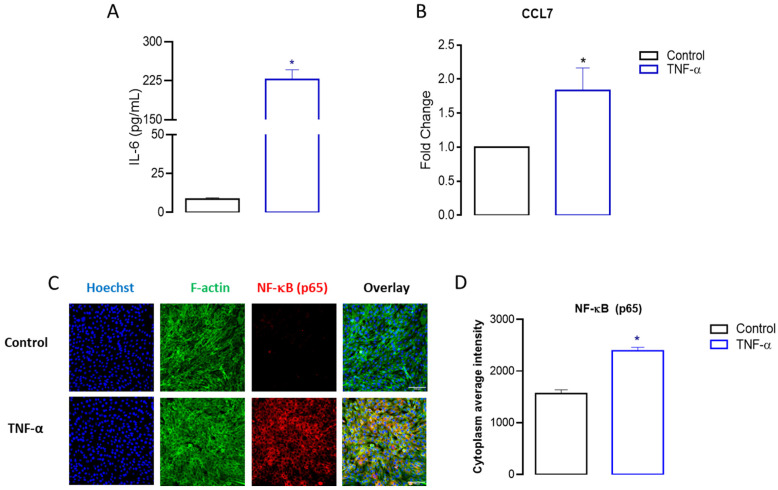
Analysis of the production of IL-6 and the expression of CCL7 and the p65 subunit of NF-KB in C2C12 cells following TNF-α incubation. The levels of IL-6, CCL7, and NF-KB were assessed using ELISA, RT-qPCR, and HCS analysis (20× magnification) in proliferative myoblast cells stimulated with TNF-α for 24 h, as described in the Materials and Methods. TNF-α stimulation led to elevated expression levels of IL-6 (**A**), CCL7 (**B**), and the p65 subunit (**C**,**D**). Relative changes were compared with the untreated control (* *p* < 0.05 from unpaired *t*-test, *n* = 3 to 4).

To further validate the RNAseq results, we conducted quantitative ELISA, RT-qPCR, and HCS experiments for IL-6, CCL7, and NF-kB (p65), respectively. According to Figure 6A, IL-6, an important myokine produced by skeletal muscle tissue, exhibited enhanced production (*p* < 0.05) in C2C12 myoblast cells incubated with TNF-α (227.5 ± 26.5 pg/mL), relative to the control group (8.5 ± 1.2 pg/mL). Additionally, the data indicate that the TNF-α incubation significantly increased gene expression of CCL7 by 45% (*p* < 0.05) compared to control cells (Figure 6B). Similarly, the results presented in Figure 6C,D demonstrate that TNF-α induces a significant increase of 35% in the expression of the p65 subunit of NF-KB, in comparison to control cells.

**Figure 7:**
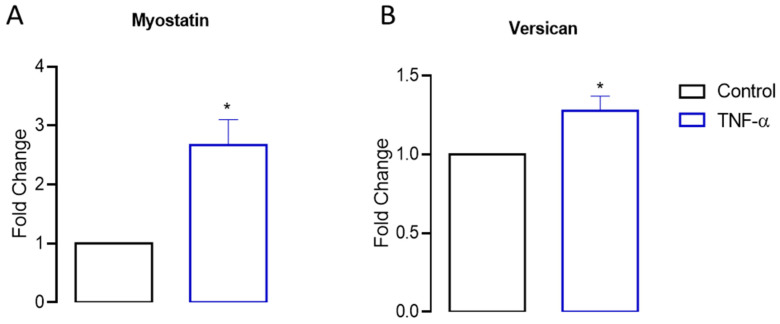
Myostatin and versican gene expressions in myoblast C2C12 cells by RT-qPCR assays. Myostatin and versican gene expressions were assessed via RT-qPCR in proliferative myoblast cells stimulated with TNF-α for 24 h, as described in the Materials and Methods. TNF-α stimulation resulted in elevated expression levels of myostatin (**A**) and versican (**B**). Relative changes in gene expressions were determined using the (2^−ΔΔCt^) method and are presented as fold changes compared to the untreated control (* *p* < 0.05 from unpaired *t*-test, *n* = 3).

To further validate the RNAseq results, we performed quantitative real-time PCR (RT-qPCR) experiments for other selected genes: (i) myostatin, involved in the proliferation regulation, and (ii) versican, involved in the extracellular matrix remodeling, since TNF-α modulated both. The results depicted in Figure 7A,B demonstrate significant upregulation (*p* < 0.05) in the gene expressions of myostatin and versican following 24 h of stimulation with TNF-α.

## 4. Discussion

Proinflammatory cytokines and chemokines expressed locally have pleiotropic effects on skeletal muscle injury, mediating both the repair and regeneration of damaged myofibers through myogenesis [12,52,57,68]. The levels of specific proinflammatory mediators secreted by and around muscle tissue significantly influence the state of activation of progenitor cells and the quality of muscle tissue regeneration. Therefore, proinflammatory cytokines and chemokines drive transcript stimulation and biosynthesis of other mediators [57,69]. One key mediator is TNF-α, which, depending on its concentration, can either propagate inflammatory responses or determine the success of tissue repair, muscle mass loss, or atrophy. Based on this knowledge, we analyzed the broad influence of TNF-α at a high concentration on gene expression in myoblast C2C12 cells during early myogenesis, which corresponds to the myoblast proliferation phase [12]. 

Our analysis of differential gene expression in myoblasts identified 954 differentially expressed genes: 326 upregulated and 628 downregulated after TNF-α incubation. Our GSEA identified seven pathways associated with cytokine production and activation in proliferating C2C12 cells, reinforcing the evidence for the complex interplay between inflammation and muscle homeostasis and previous reports of the regulation of cytokine and chemokine genes by TNF-α [70,71]. Notably, the stimulation of myoblasts by TNF-α triggered a regulatory loop on its annotated pathway as well as on the IL-17, NOD-like receptor signaling pathway, and chemokine signaling pathways in proliferating myoblasts. These novel findings amplify the understanding of the interconnected aspects that influence muscle development, regeneration, and immune responses. To our knowledge, in this study, we demonstrated the ability of TNF-α to regulate the IL-17 and NOD-like receptor (NLR) pathways in myoblasts, triggering the activation of an intricate molecular network of proinflammatory mediators. This effect may provide a muscle tissue microenvironment influencing immune cell recruitment, inflammation, and regeneration since IL-17 is pivotal in promoting inflammation within diverse inflammatory and autoimmune pathologies [72]. Although IL-17 signaling has been recognized for its ability to promote cell proliferation and tissue repair, little is known about its collaborative interplay with TNF-α in the upregulation of chemokine and cytokine genes in myogenic cells. Additionally, the NOD-like receptor (NLR) family, a group of intracellular pattern recognition receptors that serve as crucial sentinels of the innate immune system, could emerge as a mechanism by which myoblasts can initiate cell cycle entry, thereby enhancing their proliferation rate [73]. This process could result in the expression of a wide array of chemokine and cytokine genes, along with other components that are crucial for immune regulation [72]. These genes are responsible for guiding the recruitment and activation of myogenic cells, effectively shaping the microenvironment necessary for successful muscle regeneration. These new insights into the TNF-α stimulation of these signaling pathways are at the forefront of knowledge and deserve further investigation.

As commonly recognized, cytokines and chemokines play a crucial role in orchestrating immune cell recruitment, inflammation, and tissue repair. In our experimental condition, we found a notable increase in the expression of genes related to chemokines, cytokines, and other proinflammatory factors. Noteworthy increases occurred in CCL5, CCL2, CXCL3, IL-1α; IL2rg, IL1rL1, CCL7, CXCL16, CXCL10 (logFC 3.63), CXCL1 (logFC 3.57); LIF, IL6, ICAM1 (logFC 5.18), and NLRP3 (logFC 3.23). These findings are consistent with previous reports that, upon stimulation by TNF-α, proliferative myoblasts acquire an immune-like function by secreting pro-inflammatory mediators during inflammation associated with muscle injury [12,74]. Other studies identified the TNF-α-induced upregulation of CCL-2, CCL5, and IL-6 in C2C12 myotubes [28,52,75].

Cytokines and chemokines are pivotal in enhancing the inflammatory response, functioning as chemoattractants for leukocytes at the site of inflammation. Notably, CCL2, CCL5, CXCL3, CCL7, and IL-6 emerge as key mediators in this context [76]. Research findings indicate that myoblasts express receptors for the chemokines CCL2 and CCL5. Through its receptor CCR2, CCL2 (also known as monocyte chemoattractant protein-1, or MCP-1) enhances myoblast proliferation by stimulating cell cycle progression and growth. CCL-5 (also known as Regulated on Activation, Normal T Cell Expressed and Secreted, or RANTES) can boost myoblast proliferation upon binding to its receptor CCR5, triggering the intracellular pathways promoting cell growth and division [52,53,54]. By contrast, the role of CXCL3 in myogenesis is less explored compared to CCL2 and CCL5. Nevertheless, its presence in muscle tissue implies a potential contribution to muscle cell differentiation [55]. In turn, CCL7 (also known as monocyte chemotactic protein 3, or MCP-3) has recently been implicated in a potential connection to irisin, a myokine secreted by skeletal muscle cells during exercise that can stimulate myogenic cells and is associated with muscle regeneration after injury [77,78]. Consistent with our present data, we previously demonstrated that TNF-α triggers irisin production in proliferating C2C12 cells.

The crosstalk between inflammatory cytokines and cellular processes like myoblast proliferation is a critical aspect of tissue repair and regeneration. In this context, the cytokine interleukin-1α (IL-1α) and its induction by TNF-α can significantly influence myoblast proliferation through both autocrine (acting on the producing cell) and paracrine (affecting neighboring cells) mechanisms [79,80]. In turn, the cytokine IL-6, secreted by muscle cells, initiates myogenesis by promoting myoblast proliferation and activation [7,12,57,71]. Thus, our findings showing an increased expression of IL-1α, and IL-6 allow us to suggest that low concentrations of TNF-α can induce myoblast proliferation and activation. In agreement with this hypothesis, our previous study revealed a clear association between the TNF-α-induced proliferation of C2C12 cells and IL-6 receptor activation [12].

The transcription factor NF-κB (Nuclear Factor Kappa B) plays a pivotal role in regulating various cellular processes, including cell proliferation. Studies have demonstrated that, when stimulated by TNF-α, C2C12 cells activate the NF-κB pathway, leading to the transcription of genes crucial for muscle tissue regeneration [7,28,81]. NF-κB serves as a key regulator, activating the expression of genes associated with the cell cycle, such as cyclins and cyclin-dependent kinases [82]. Furthermore, NF-κB can regulate the expression of growth factors and their receptors, as well as the proteins involved in cell adhesion and intercellular communication, all of which are essential for cell proliferation [83]. Additionally, upon NF-κB activation, chemokines such as CCL2 and CCL3, along with the cytokine IL-6, are secreted, coordinating the recruitment of inflammatory cells to the injury site while also modulating the activity of myogenic cells [28,84,85]. This coordinated response creates an environment conducive to cell proliferation by providing additional signals that enhance cell survival and division.

Research findings indicate that TNF-α promotes myoblast proliferation via JNK1 while preventing myoblast differentiation by triggering the secretion of the myokine LIF (leukemia inhibitory factor), an IL-6 family member—through JNK1-mediated pathways [86]. This aspect is intriguing, since existing studies have highlighted that TNF-α can either promote proliferation or hinder the transition of myogenic cells towards premature differentiation [7,26]. Based on that and considering that LIF enhances myoblast proliferation [58,87,88], our results showing that TNF-α up-modulates LIF in myoblasts suggest that LIF constitutes an inhibitory factor in differentiation following TNF-α stimulation. Another myokine renowned for its role in inhibiting and downregulating the process of myoblast differentiation is myostatin [89]. Although our earlier research confirmed that TNF-α does not impact the secreted myostatin levels, we assessed its expression via RT-qPCR in the presence of the TNF-α effect [12]. To our knowledge, our findings reveal for the first time that TNF-α induces an increased gene expression of myostatin, providing evidence of its negative regulatory effect on myoblast proliferation through the up-modulation of that gene expression. This reinforces TNF-α’s inhibitory role in differentiation since myostatin down-modulates myoblast proliferation by dampening the expression of pivotal myogenic regulatory factors (MRFs), such as MyoD and myogenin [90]. These key transcription factors orchestrate differentiation and muscle fiber formation [65]. 

To validate the regulatory effects of the low concentrations of TNF-α on myoblast proliferation and differentiation, we examined its influence on muscle-specific transcription factors controlling myogenesis stages. Our findings reveal that TNF-α leads to an increase in the protein expression of myogenic factor 5 (Myf5) while simultaneously reducing the protein levels of MyoD. During the initial stages of myoblast culture, before differentiation occurs, Myf5 influences the cell cycle machinery, ensuring a balanced progression through the cell cycle stages that are necessary for cell proliferation [91]. In addition, Myf5 contributes to the process moving forward, culminating in fusion and differentiation into myotubes [92].

Our analysis also identified other inflammatory genes upregulated by TNF-α in proliferating myoblasts, including Intercellular Adhesion Molecule-1 (ICAM-1). Inflammation is a hallmark of tissue injury and regeneration. TNF-α-induced ICAM-1 expression contributes to immune cell recruitment, creating an environment that impacts myoblast behavior [78,93]. As a cell surface glycoprotein, ICAM-1 enables immune cell adhesion and migration. When expressed in muscle cells, it promotes injury recovery by orchestrating mechanisms that regulate myofiber branching, protein synthesis, and the precise myonuclei organization after myogenic cell fusion [94]. These reports are in line with our findings, which demonstrate the up-regulation of genes for the chemotactic chemokines CCL-2, CCL5, CXCL3, CCL7, and the cytokine IL-6, allowing us to suggest that low concentration of TNF-α facilitates muscle regeneration by inducing leukocyte infiltration, among the main mechanisms involved. 

Our data show for the first time the ability of TNF-α to modulate NLRP3 (Nucleotide-binding domain, Leucine-rich Repeat, Pyrin domain-containing protein 3) in proliferative myoblasts. NLRP3 is an important NLR and a component of the innate immune system, primarily known for its role in inflammasome activation and positive regulation of inflammation [95]. Although its role in immune responses has been extensively studied, the direct influence of NLRP3 on myoblast proliferation and muscle regeneration has garnered less attention. Nonetheless, emerging evidence points towards its potential contribution to inflammation-related skeletal muscle wasting [96]. Such studies are in agreement with our results since the NOD-like receptor pathway was significantly enriched, as revealed by the gene set enrichment analysis. Although this pathway deserves our attention and further research, we focused on the other pathways based on the ample evidence regarding their role in skeletal muscle development.

Interestingly, the transcriptomic analysis of TNF-α-treated C2C12 cells also revealed the marked up-regulation of MMPs, including MMP-9 (logFC 7.72), MMP-10 (logFC 7.14), and MMP-13 (logFC 8.84). These MMPs play key roles in physiological and pathological processes, including myoblast proliferation during myogenesis. Accumulating evidence shows that MMP-mediated extracellular matrix proteolysis aids myogenesis by degrading components like collagen and fibronectin, leading to expanding myoblasts to migrate and proliferate [97,98,99,100]. Studies have shown that myogenic cells, when activated after tissue injury, can produce active MMPs, stimulating cell migration to the injured site [27,66]. This links myogenic cell activation, MMP production, and regenerative myogenesis with proinflammatory cytokine release after injury. Some authors suggest that TNF-α can stimulate the migration of myoblasts to the site of tissue injury and induce regeneration through MMP-mediated chemotaxis and the secretion of chemotactic mediators and adhesion molecules [22,101]. Moreover, MMP-9, MMP-10, and MMP-13 can also influence myoblast proliferation by modulating the activity of growth factors and other cytokines. They can cleave and activate growth factors such as transforming growth factor beta (TGF-β) and insulin-like growth factor (IGF), which are known to promote myoblast proliferation and differentiation [67,101]. Considering the above information, our results suggest that low-concentration TNF-α is associated with increased myoblast proliferation. However, future studies are needed to validate our hypothesis. It must be pointed out that TNF-α’s regulation of MMPs during myoblast proliferation requires tight control to prevent excessive ECM degradation and aberrant cell behaviors, as the dysregulation of these MMPs can lead to impaired myoblast proliferation and disrupted muscle tissue development [102]. Such control may be led by the inhibitor TIMP-1, since we found increased expression at the protein level induced by the TNF-α treatment. It would be a compensatory mechanism since myoblast migration is associated with low MMP-9 but overexpressed MMP-2 and TIMP-1 [103]. Although the TIMP-1 expression is frequently associated with MMP-9 inhibition instead of other MMPs [104], we did not find a reduction in MMP-9 levels. Therefore, future studies are needed to validate our hypothesis.

## 5. Conclusions

In summary, our study sheds light on the effects of TNF-α, a key proinflammatory mediator, on myoblasts during myogenic proliferation. We demonstrate here that TNF-α leads to a complex cascade of events involving the TNF-α, IL-17, chemokine signaling and NOD-like receptor signaling pathways. These interconnected pathways contribute to the regulation of cytokine and chemokine gene expression, ultimately influencing the immune cell recruitment, inflammation, and tissue regeneration. Our transcriptomic analysis confirmed TNF-α’s role in promoting myoblast proliferation by increasing the expression of (1) relevant chemokines, such as CCL-2, CCL-5, CCL-7, and CXCL3, (2) relevant cytokines, such as IL1-α, and (3) relevant myokines, including IL-6 and LIF. Collectively, these mediators can modulate crucial myogenic transcription factors, notably Myf5, directing myoblasts toward increased proliferation. Concurrently, TNF-α upregulates the gene expression of myostatin, an essential myokine that plays a regulatory role by reducing the process of myoblast differentiation. Furthermore, we provide novel insights into TNF-α’s influence on MMP expression, particularly MMP-9, MMP-10, and MMP-13, which contribute to extracellular matrix remodeling, myoblast migration, and growth factor activation. Collectively, our data underscore the complexity of the impact of a high concentration of TNF-α on myoblast proliferation and its significant role in orchestrating the inflammatory microenvironment necessary for effective muscle tissue repair and regeneration. Despite these findings, we acknowledge the limitations of our study in fully validating all the characterized pathways influenced by TNF-α. Additionally, further research is needed to explore the pathways mediated by cytokines and chemokines that modulate myogenic cells during differentiation. It is also crucial to correlate these pathways with those induced by TNF-α during the initial regeneration process, specifically cell proliferation. Future studies can build on these findings to elucidate the in vivo relevance of the identified signaling pathways and gene networks modulated by TNF-α. Therapeutic strategies targeting the components of these pathways could then be explored to enhance muscle regeneration after injury.

## Figures and Tables

**Table 1:** RT-qPCR primers used in this study.

Gen Name	Primer Sequence
Myostatin	F: ATGGCAAGCCCAAATGTTGC R: AGGAGTCTTGACGGGTCTGA
Versican	F: ACCAAGGAGAAGTTCGAGCA R: CTTCCCAGGTAGCCAAATCA
CCL7	F: GCTGCTTTCAGCATCCAAGTG R; CCAGGGACACCGACTACTG
GAPDH	F: AGGTCGGTGTGAACGGATTTG R: TGTAGACCATGTAGTTGAGGTCA

**Table 2:** GSEA results. The “description” column shows the enriched KEGG pathways; “Set size” represents the number of genes in the gene set; “Enrichment score” is the degree to which this gene set is overrepresented at the top (upregulated) or bottom (downregulated) of the ranked list of genes in the expression dataset; “NES”, the normalized enrichment score, is the enrichment score for the gene set after it has been normalized across analyzed gene sets; “*p*-value” is the statistical significance of the enrichment score; “FDR”, the false discovery rate, is the adjusted *p*-value for repetitive comparisons; “rank” means the position in the ranked list at which the maximum enrichment score occurred; “Leading edge” shows the leading edge analysis results, used to analyze the overlap between multiple leading-edge subsets; “Genes” are the DEGs found within the gene set.

Description	Set Size	Enrichment Score	NES	*p*-Value	FDR	Rank	Leading Edge	Core Enrichment
TNF signaling pathway	93	0.8	2.74	1.00 × 10^−10^	1.43 × 10^−8^	614	tags = 31%, list = 4%, signal = 30%	CCL5/MMP9/BIRC3/CCL2/TRAF1/CXCL3/ICAM1/CXCL10/CXCL1/LIF/IL6/NFKBIA/TNFAIP3/BCL3/FAS/VCAM1/PTGS2/CEBPB/IRF1/CX3CL1/IL15/JUNB/NOD2/TRAF3/MAP3K8/EDN1/MAPK11/MAP3K5/TRAF2
IL−17 signaling pathway	61	0.79	2.55	1.00 × 10^−10^	1.43 × 10^−8^	614	tags = 30%, list = 4%, signal = 28%	MMP13/MMP9/CCL2/CXCL3/CCL7/CXCL10/CXCL1/CXCL5/IL6/NFKBIA/TNFAIP3/CCL11/PTGS2/CEBPB/FOSL1/TRAF3/MAPK11/TRAF2
NOD-like receptor signaling pathway	119	0.67	2.42	2.10 × 10^−10^	1.99 × 10^−8^	1244	tags = 24%, list = 9%, signal = 22%	CCL5/BIRC3/CCL2/CXCL3/GBP2/GBP3/GBP2B/CXCL1/NLRP3/IL6/NFKBIA/TNFAIP3/CASP4/CAMP/NOD2/TRAF3/GSDMD/RIPK2/NFKBIB/MAPK11/TRAF2/ITPR1/AIM2/CASP1/TANK/MYD88/DHX33/NFKB1/HSP90AA1
Chemokine signaling pathway	114	0.63	2.26	1.18 × 10^−08^	8.41 × 10^−7^	381	tags = 14%, list = 3%, signal = 14%	CCL5/CCL2/CXCL3/CCL7/CXCL16/CXCL10/CXCL1/PIK3R5/CXCL5/CCL9/NFKBIA/CCL8/CCL11/CXCR6/CX3CL1/JAK2

## Data Availability

Data are contained within the article and Supplementary Materials.

## Supplementary Materials

The following supporting information can be downloaded at: https://www.mdpi.com/article/10.3390/cells13131161/s1. Table S1: DEGs TNF vs. CTRL and Table S2: Gene set enrichment analysis and KEGG.
